# Delusional Severity Is Associated with Abnormal Texture in FLAIR MRI

**DOI:** 10.3390/brainsci12050600

**Published:** 2022-05-05

**Authors:** Marc A. Khoury, Mohamad-Ali Bahsoun, Ayad Fadhel, Shukrullah Shunbuli, Saanika Venkatesh, Abdollah Ghazvanchahi, Samir Mitha, Karissa Chan, Luis R. Fornazzari, Nathan W. Churchill, Zahinoor Ismail, David G. Munoz, Tom A. Schweizer, Alan R. Moody, Corinne E. Fischer, April Khademi

**Affiliations:** 1 Keenan Research Centre for Biomedical Science, Li Ka Shing Knowledge Institute, Toronto, ON M5V 1T8, Canada; marc.khoury@unityhealth.to (M.A.K.); afadhel@my.yorku.ca (A.F.); shakur.shunbuli@gmail.com (S.S.); saanikavenkatesh@gmail.com (S.V.); luis.fornazzari@gmail.com (L.R.F.); nchurchill.research@gmail.com (N.W.C.); David.Munoz@unityhealth.to (D.G.M.); Tom.Schweizer@unityhealth.to (T.A.S.); akhademi@ryerson.ca (A.K.); 2 Institute for Biomedical Engineering, Science & Tech (iBEST), a Partnership between St. Michael’s Hospital and Ryerson University, Toronto, ON M5V 1T8, Canada; mbahsoun@ryerson.ca (M.-A.B.); aghazvan@ryerson.ca (A.G.); samir.mitha@ryerson.ca (S.M.); karissa.chan@ryerson.ca (K.C.); 3 Electrical, Computer and Biomedical Engineering Department, Ryerson University, Toronto, ON M5B 2K3, Canada; 4 Institute of Health Policy Management and Evaluation, University of Toronto, Toronto, ON M5T 3M6, Canada; 5 Division of Neurology, Faculty of Medicine, University of Toronto, Toronto, ON M5S 3H2, Canada; 6 Institute of Medical Sciences, University of Toronto, Toronto, ON M5S 1A8, Canada; 7 Departments of Psychiatry, Clinical Neurosciences, and Community Health Sciences, Hotchkiss Brain Institute, University of Calgary, Calgary, AB T2N 4N1, Canada; ismailz@ucalgary.ca; 8 Department of Laboratory Medicine and Pathobiology, University of Toronto, Toronto, ON M5S 1A8, Canada; 9 Institute of Biomaterials and Biomedical Engineering, University of Toronto, Toronto, ON M5S 3G9, Canada; 10 Division of Neurosurgery, Department of Surgery, Faculty of Medicine, University of Toronto, Toronto, ON M5T 1P5, Canada; 11 Department of Medical Imaging, University of Toronto, Toronto, ON M5T 1W7, Canada; Alan.moody@sunnybrook.ca; 12 Department of Psychiatry, Faculty of Medicine, University of Toronto, Toronto, ON M5T 1R8, Canada

**Keywords:** delusions, white-matter texture, biomarkers, Alzheimer’s disease, dementia, cognitive decline, CSF tau, APOE4

## Abstract

Background: This study examines the relationship between delusional severity in cognitively impaired adults with automatically computed volume and texture biomarkers from the Normal Appearing Brain Matter (NABM) in FLAIR MRI. Methods: Patients with mild cognitive impairment (MCI, *n* = 24) and Alzheimer’s Disease (AD, *n* = 18) with delusions of varying severities based on Neuropsychiatric Inventory-Questionnaire (NPI-Q) (1—mild, 2—moderate, 3—severe) from the Alzheimer’s Disease Neuroimaging Initiative (ADNI) were analyzed for this task. The NABM region, which is gray matter (GM) and white matter (WM) combined, was automatically segmented in FLAIR MRI volumes with intensity standardization and thresholding. Three imaging biomarkers were computed from this region, including NABM volume and two texture markers called “Integrity” and “Damage”. Together, these imaging biomarkers quantify structural changes in brain volume, microstructural integrity and tissue damage. Multivariable regression was used to investigate relationships between imaging biomarkers and delusional severities (1, 2 and 3). Sex, age, education, APOE4 and baseline cerebrospinal fluid (CSF) tau were included as co-variates. Results: Biomarkers were extracted from a total of 42 participants with longitudinal time points representing 164 imaging volumes. Significant associations were found for all three NABM biomarkers between delusion level 3 and level 1. Integrity was also sensitive enough to show differences between delusion level 1 and delusion level 2. A significant specified interaction was noted with severe delusions (level 3) and CSF tau for all imaging biomarkers (*p* < 0.01). APOE4 homozygotes were also significantly related to the biomarkers. Conclusion: Cognitively impaired older adults with more severe delusions have greater global brain disease burden in the WM and GM combined (NABM) as measured using FLAIR MRI. Relative to patients with mild delusions, tissue degeneration in the NABM was more pronounced in subjects with higher delusional symptoms, with a significant association with CSF tau. Future studies are required to establish potential tau-associated mechanisms of increased delusional severity.

## 1. Introduction

Delusions are false beliefs sometimes experienced by older people that are associated with adverse clinical outcomes such as cognitive impairment [1], accelerated cognitive decline, premature institutionalization and increased conversion to dementia [2,3]. Delusions can occur across the spectrum of cognitive impairment, with a 30% prevalence in patients with Alzheimer’s disease (AD) [2]. Despite the prevalence and adverse clinical outcomes of delusions, their mechanisms remain unclear [4].

Neuroimaging studies have investigated brain structure changes in magnetic resonance imaging (MRI) associated with delusions, but were largely inconclusive [5]. Rootes-Murdy et al. [5] examined gray matter (GM) structures among patients with delusions across multiple disorders including bipolar disorder, schizophrenia, AD and Parkinson’s disease, and found relative atrophy in a number of brain regions including the dorsolateral prefrontal cortex, left claustrum, hippocampus, insula, amygdala, thalamus, superior temporal gyrus, and middle–frontal gyrus. However, the affected brain regions differed considerably by disorder. Cross-sectional studies reported decreased GM volume in AD patients with delusions relative to patients without delusions, predominantly in the right frontal cortex [6,7]. Longitudinal studies among patients with delusions and AD by our group have shown accelerated GM atrophy in the frontal and temporal lobes preceding onset of delusions relative to a non-delusional cohort [8]. Nakkaki et al. found reduced white matter integrity in the left parieto-occipital region and corpus callosum when comparing AD patients who develop delusions to those who did not [9]. Lee et al. found a correlation between the misidentification delusions and white matter integrity without infarcts or lacunae in the frontal, right parieto-occipital and left basal ganglia, though no significant correlations were found with other behavioral domains [10].

The presence of distributed changes in GM associated with psychosis may be due to WM disease, which disrupts networks in the brain. This has been well-examined in the literature, particularly among psychotic disorders such as schizophrenia [11], where diffusion tensor imaging (DTI) techniques demonstrated widespread disruption of WM tracts [11]. The mechanism underlying WM disruption in patients with neurodegenerative disorders suffering delusions is not yet clear, though studies suggest that tau plays a putative role both in delusions [12] and as a disrupter of regional white matter [13]. Tau is a microtubule stabilizer in neuronal axons [14] and is associated with neuronal death [15]. Tau can be used to assess risk of developing dementia [16], and its effects on neurodegeneration and delusions are investigated in this work.

Apolipoprotein E4 (APOE4 heterozygous), a known genetic risk factor for AD, may also contribute to delusion formation in patients with neuropathologically confirmed AD, in particular among female APOE4 homozygotes, based on data from our group [17]. Whilst the exact relationship between delusions and APOE4 is unclear, APOE4 has been associated with increased levels of delusions in early stages of Alzheimer’s disease with late onset [18]. It also been shown that patients with the APOE E4 allele possess greater tau accumulation and brain atrophy in the medial temporal lobe, contributing to overall memory impairment [19,20] relative to APOE4-negative patients, who may have greater accumulation of tau in the frontal and parietal lobes, contributing to greater executive dysfunction. We investigate the effect of APOE status on neurodegeneration in subjects with delusions. 

While most studies examine microstructural changes using DTI metrics, in this work, we focus on texture biomarkers from the Normal-Appearing Brain Matter (NABM) in FLAIR MRI to quantify microstructural changes in the brain. The NABM is gray matter (GM) and white matter (WM) combined, i.e., brain tissue with cerebrospinal fluid and white matter lesions (WML) removed (see Table A3). Studies to date suggest a correlation between delusions and WML [8,9,10,11], but they have not yet examined the correlation between delusions and structural changes in the GM and WM combined. NABM texture biomarkers in FLAIR MRI are related to microstructural brain tissue changes in cognitive populations and can distinguish between patients with varying levels of cognitive impairment ranging from cognitively normal to AD [21]. NABM texture refers to tissue organization and offers a complementary perspective with promise in measuring early stage disease [21]. Although interest in MRI texture analysis is growing [22], there are limited studies in subjects with neurodegenerative disorders and delusions. 

In this study, we investigate FLAIR MRI NABM texture and volume of MCI and AD patients extracted from the Alzheimer’s Disease Neuroimaging Database (ADNI) who had delusions of varying severities. We hypothesized that severity of delusions would be associated with more severe texture abnormalities in the NABM given the association of psychotic symptoms with abnormal changes in the GM and WM, and that tau and APOE4 (heterozygote) may play an important modulatory role. Clarifying how delusional severity relates to NABM texture or volume may identify underlying mechanisms, serve as a surrogate marker to guide treatment and aid in the detection of subvisual cues related to disease.

## 2. Materials and Methods

### 2.1. Study Design and Participants

All research procedures were approved by the St. Michael’s Hospital Research Ethics Board in accordance with ethical standards laid down in the 1964 Declaration of Helsinki and its later amendments. Participant data were obtained from the Alzheimer’s Disease Neuroimaging Initiative (ADNI) database (adni.loni.usc.edu, accessed on 1 January 2016), which was launched in 2003 as a public–private partnership, led by Principal Investigator Michael W. Weiner, MD. The primary goal of ADNI is to test whether serial magnetic resonance imaging (MRI), positron emission tomography (PET), other biological markers, and clinical and neuropsychological assessment can be combined to measure differences between mild cognitive impairment (MCI) and Alzheimer’s disease (AD). For up-to-date information, see www.adni-info.org (accessed on 1 January 2016). For this study, data from ADNI 2 and ADNI GO were used and subjects without FLAIR imaging or Neuropsychiatric Inventory-Questionnaire (NPI-Q) data were excluded. We included patients who had MCI and those with AD, given the potential overlap between these two cognitive states and to maximize sample size. In total, 42 patients with delusions were extracted from ADNI 2, 24 having MCI, 17 having AD and 1 converting from MCI to AD with delusions. There was one patient from ADNIGO with MCI. In total, there were 42 subjects with longitudinal time points resulting in 164 imaging volumes (approximately 5400 image slices) with 112 imaging volumes in group 1 (low delusional severity), 7 subjects (30 imaging volumes) in group 2 (intermediate delusion severity) and 4 subjects (22 imaging volumes) in group 3 (high delusion severity). Six subjects had multiple severity scores including one with severities 1, 2 and 3, one with severities 1 and 3, two with severities 1 and 2 and two with severities 2 and 3 (see Table A2). Patients with multiple delusional severities were included, given that each patient encounter had a different MRI scan that corresponded to that severity. 

### 2.2. Behavioral Analyses

Delusions were identified using the Neuropsychiatric Inventory Questionnaire [23] (NPI-Q), with severity ranging from 1 to 3. An NPI-Q score of 1 is the lowest severity (reference) and 3 is the highest severity. The NPI-Q measures the presence and severity of neuropsychiatric symptoms (NPS) and includes 12 domains: delusions, hallucinations, agitation/aggression, depression/dysphoria, anxiety, elation/euphoria, apathy/indifference, disinhibition, irritability/lability, motor disturbance, night-time behavior and appetite disturbance. Each behavior is rated on a scale of 1 to 3, with 1 being the least severe and 3 the most severe. The individual behaviors are summed to find the total NPI-Q score. 

### 2.3. APOE and CSF Markers

APOE status was available for APOE4 homozygotes (E44) subjects (*n* = 9) (38 imaging volumes), APOE4 heterozygotes (E4) subjects (*n* = 14) (63 imaging volumes), and APOE4 non-carrier (NN) subjects (*n* = 18) (63 imaging volumes). Cerebrospinal fluid (CSF) tau was available for 151 samples. 

### 2.4. Imaging Analyses

FLAIR imaging volumes were downloaded for each subject. The acquisition parameters for this cohort are shown in Table A1. Images were acquired with GE/Siemens/Philips scanners from 26 imaging centers worldwide, representing a large multicenter dataset. A validated intracranial volume (ICV) extraction tool for multicenter FLAIR MRI [24] was used for skull stripping to remove irrelevant tissue. Intensity standardization was then applied to align the intensity distribution of the subjects’ images to an atlas [22] and images were resampled to a resolution of 0.85 mm × 0.85 mm × 3 mm. Intensity normalization and spatial resampling helps to reliably compare biomarkers across subjects in multicenter data [22]. The NABM of each volume was segmented by stripping out lesions and CSF through thresholding the intensity-standardized images [22,24]. The NABM is the GM and WM tissue regions combined, and since neurodegeneration can affect both GM and WM, we analyze changes from both these regions. 

**Figure 1:**
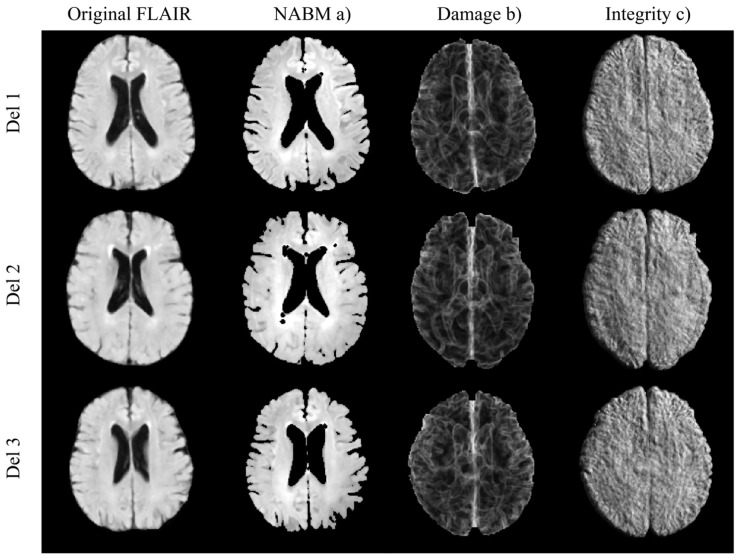
FLAIR MRI slices for original, NABM (**a**) and Damage (**b**) and Integrity markers (**c**).

From the NABM region, three biomarkers were measured per imaging volume, as described in [21]: NABM volume, microstructural integrity (Integrity) and macrostructural damage (Damage). The NABM volume is the total volume of GM and WM together, normalized by the ICV and is computed based on the number of pixels in the region and voxel resolution. The integrity and damage biomarkers were derived using 2D texture analysis [21] that measures changes in intensities in small windows. A 5 × 5 square window was used for texture analysis, which corresponds to a spatial resolution of 4.25 mm × 4.25 mm. For each slice, a texture feature was extracted per pixel that measures the local variation in intensities within each window, and the resultant 2D texture maps were averaged pixel-wise across the 3D volume of the brain. The result is a mean texture image describing the global texture of the brain per subject. The median of this feature map is used as the biomarker. An example of the 2D map for integrity and damage are shown in Figure 1. A higher integrity value represents more repeating edge patterns which indicates less randomness in the microstructure. A higher value for the damage marker indicates more intensity discontinuities corresponding to neurodegeneration. Since NABM texture features quantify changes and variance in intensities (related to texture), we postulate that texture features quantify global disease burden related microstructural damage and structural integrity in the NABM. Multivariable and stepwise regression analysis were completed to examine differences in imaging biomarkers across delusional scores.

### 2.5. Statistical Analyses

To examine the association between delusions and NABM biomarkers (volume, integrity and damage), the following multivariable regression model is used:

NABM ~ Delusions + Tau + APOE4 + Age + Sex + Education + Tau*Delusions(1)

NABM imaging biomarkers are the outcome (dependent) variables and delusion scores 1, 2 and 3, along with covariates are the independent variables. Covariates included CSF tau, APOE (NN, E4 and E44), age, sex and education. An interaction term was added to investigate the relationship between delusional severity and CSF tau. *Stepwise regression* was used to examine model robustness. Significance was assessed with a confidence interval of 95%. Results for categorical variables are reported with reference to the lowest level (i.e., significant differences in biomarkers between delusion score 1 vs. delusion score 2 or delusion score 3). 

The NPI-Q Delusion scores of “*1-Mild*” delusions are considered less severe than “*2-Moderate*” delusions and “*3-Severe*” delusions. Thus, moderate and severe delusions were merged into a single “Severe Delusions” group that is compared to the “Mild Delusions” group in a post hoc analysis. The same model as above was included to investigate differences in biomarkers between mild delusions (score 1, *112 imaging volumes*) and severe delusions (2—moderate delusions + 3—severe delusions combined, *53 imaging volumes*). Multiple comparisons was used to determine differences in biomarker means between mild and severe delusions. 

## 3. Results

NABM volume and texture (integrity and damage) imaging biomarkers were extracted from each FLAIR MRI volume. Imaging biomarkers were collected and saved for statistical analysis, along with clinical variables related to delusional severity, APOE and baseline CSF tau (320.0 ± 157.7). The mean age (74.5 ± 8.3 years), mean sex and mean delusional severity for the three diagnostic groups (MCI, AD, MCI transitioning to AD) are provided in Table A2. Stepwise regression demonstrated no forced-in or forced-out values for the regression model, confirming that model produces robust predictions.

### 3.1. Multivariable Regression

**Figure 2:**
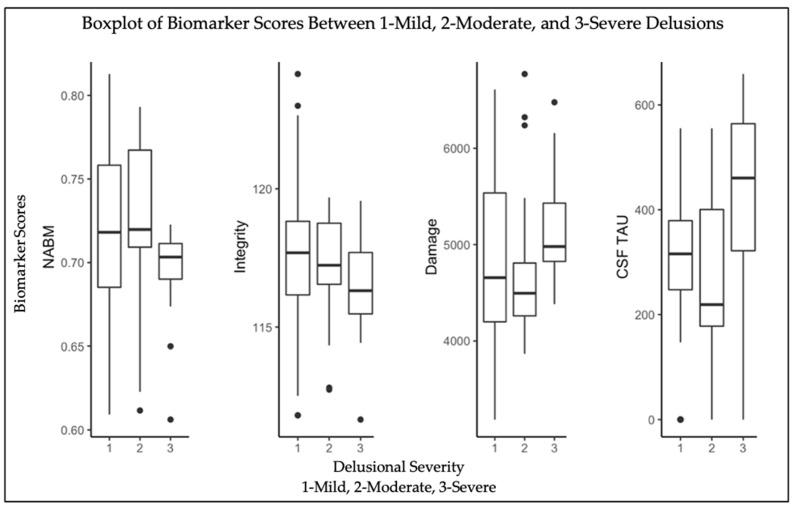
Distributions of NABM volume, integrity, and damage biomarkers from FLAIR, as well as CSF tau between 1—mild delusions, 2—moderate delusions, 3—severe delusions.

Results from the multivariable regression model in Equation (1) for all three delusion levels and each NABM biomarker is shown Table 1. Analysis reveals significance for delusional severity group 3 compared to delusion severity group 1 across all three imaging biomarkers, the lowest *p*-value being for NABM volume (*p* < 0.05; see Table 1). Delusion severity group 2 compared to delusion severity group 1 was significant for only the integrity biomarker (*p* < 0.05). NABM biomarker distributions across imaging volumes in Figure 2 reveal decreasing NABM volume and integrity for higher delusional scores with integrity exhibiting the most notable differences between delusion severity groups. There is an increasing trend for the damage biomarker values for higher levels of delusions, although delusion level 1 has a wider variance, with more similar means compared to delusion level 2. It is also noted that CSF tau is higher for the higher NPI-Q delusional scores.

The linear regression coefficients for each variable are shown in Table 1 as well, which represents the contribution of each variable. The NABM volume is a percentage and shows the difference in normalized GM+WM volumes between groups. There is reduced volume for higher delusions, with a marked difference between delusion 1 and delusion 3 (=9.4%). The integrity variable has a small range, but as previously found [21], due to intensity standardization and robust texture representation, small differences are significant across groups, as was further supported in this work. As noted, there is reduced integrity for higher delusions compared to delusion 1 (=−3.71). The damage biomarker examines spatial correlation between neighboring pixels by measuring the square difference of pixel intensities in the neighborhood. As a result, this biomarker has a wider range (and a higher valued), as was also confirmed [21]. As is shown, there is more damage (higher value) for this marker compared to delusion 1 (=927.90).

Regarding covariates, all biomarkers were significantly dependent on sex and age (*p* < 0.01), whereas education level did not play a significant role. Baseline CSF tau’s main effects were significantly associated with NABM volume and integrity, but not damage (see Table 1). The association between integrity and CSF tau appears to be stronger in comparison to NABM and CSF tau given the lower *p*-value. Interestingly, APOE44 (homozygotes) were significantly associated with all biomarkers. When specifying for interactions, baseline CSF tau and delusion score 3 were significantly correlated across all biomarkers. Baseline CSF tau had a greater median value for delusions 3 and a lower median value for less severe delusions (see Figure 2).

### 3.2. Post Hoc Analysis

**Figure 3:**
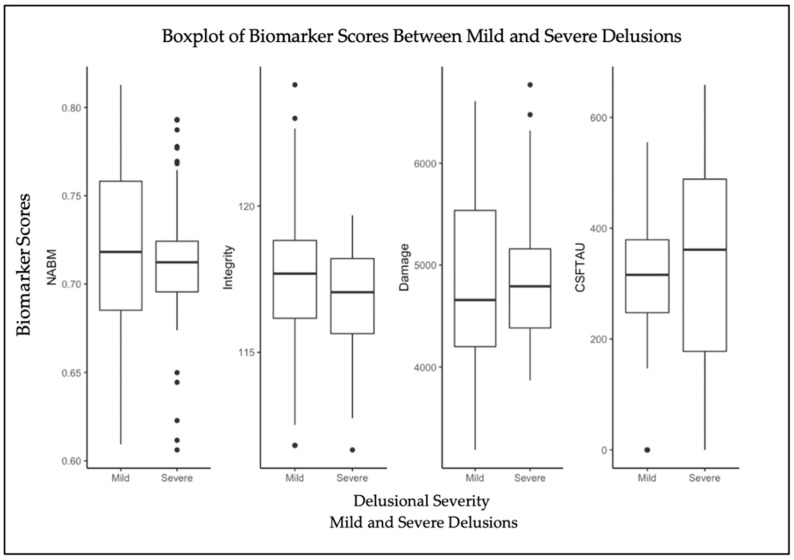
Distributions of NABM volume, integrity, and damage biomarkers from FLAIR, as well as CSF tau between mild delusions and severe delusions.

To examine differences between biomarkers between “Mild Delusions” and “Severe Delusions”, the multivariate regression model (Equation (1)) was applied to the two delusions groups. Statistical results are reported in Table 2 and biomarker distributions are shown in Figure 3. There are significant differences in NABM volume and integrity markers across mild- and severe-delusion groups, but not for damage. For the severe-delusions group, NABM volume (*p* = 5.48 × 10^−3^) and integrity (*p =* 7.26 × 10^−4^) have significantly lower means, with integrity showing the most noticeable difference between the mild- and severe-delusions groups (Table 2). The damage marker is higher for severe delusions, although the difference between delusional groups was not significant. As shown in Figure 2, which is based on three delusion groups, the variance in the damage biomarker is high for delusion group 1, and biomarker means are more similar between delusion 1 and 2, which could explain these results. CSF tau was significant across all biomarkers (Table 2), as were sex and age. Similarly to in the three-delusion-group analysis, there was no association with education level, but all three biomarkers were significantly associated with APOE44 (homozygotes). With the exception of damage, all biomarkers had significant interactions between delusions and CSF tau.

## 4. Discussion

Our findings suggest abnormalities in normal-appearing brain matter (NABM) in FLAIR MRI are associated with severity of delusions in cognitively impaired older adults. Specifically, in subjects with greater delusional severity (NPI-Q severity score of 3 alone as well as 2 + 3 combined), the integrity and NABM volume biomarkers were lower and damage biomarkers were higher for more severe delusions. The texture biomarkers describe local intensity variations in tissue microstructure and show that texture was more random with less “structure” when compared to patients with less-severe delusions (NPI-Q severity score of 1). This was true of all markers. Integrity was the only marker more sensitive to subtle differences in delusional severity, showing differences between NPI-Q severity scores 2 and 1, 3 and 1 as well as the two-group analysis between mild and severe delusions. This may indicate there are more noticeable changes in the GM + WM texture patterns over all delusional groups, as elucidated by the integrity biomarker. Therefore, integrity may be a more sensitive marker of delusional severity than volume and damage, which could possibly indicate that integrity (texture) changes in the GM and WM precede the onset of volumetric changes which are associated with progression from mild to severe delusions. Integrity is based on local-binary patterns, and higher values are related to edge or high-frequency patterns throughout the brain that are repeating and have the same microstructural patterns, which is indicative of healthier tissue [21]. 

As for the damage marker, while it did not show significant differences in the two-delusions-group analysis, it does show that for higher delusions there is more damage. The damage marker is based on the analysis of intensity differences in local windows, with regions that have more discontinuities having a higher value. Therefore, in regions where texture is more random, or “rough”, there is higher damage, which was found for more severe delusions. It was shown that NABM textures in FLAIR MRI are correlated with water diffusion in DTI [21]; although subtle differences were noted and more investigation into the contrast mechanisms in FLAIR MRI will be part of future work. However, it is known that FLAIR measures ischemia, edema and is related to lipid attenuation in axons—which are related to disease processes in the brain. 

The NABM volume biomarker also showed significant differences between delusions 1 and 3, as well as for the two-group analysis between mild and severe delusions. As NABM is the GM and WM combined, this demonstrates that neurodegenerative processes and delusions affect both the GM and WM. In addition to this, our findings indicate NABM volumes (GM + WM) of NPI-Q 1 and NPI-Q 2 are more similar (i.e., the amount of tissue loss in the GM and WM is roughly similar in these delusional groups), and so lower severity delusions may not elicit significant alterations in the NABM volume. However, there was overlap in the biomarkers between NPI-Q 1 and NPI-Q 2 delusion scores, as shown by the biomarker distributions and the statistical analysis. The assessment of delusional severity is often subjective in nature, and thus clinically there may be only marginal differences between delusional severities 1 and 2.

Prior studies focused on older patients with delusions have suggested that WM disease may be associated with late-life psychosis, in particular among patients with cognitive disorders such as AD [4,10]. Gray matter changes have also been noted among AD patients with delusions relative to those without, in particular, localized to the right frontal cortex [6,7]. Longitudinal GM changes have also been found to precede onset of delusions, in particular in the frontal and temporal lobes [8]. A potential theory is that damage to WM structures in patients with psychotic disorders may ultimately lead to GM damage [11], although this is still being investigated within the community. However, it is clear that delusional processes do affect both the WM and GM structures, and as a result, measuring the changes in the NABM offers a single metric for global disease burden related to delusions. NABM volume changes indicate that the GM atrophy and WM loss are related to delusions. In terms of NABM texture, there is more randomness (and less structure) for higher delusional severity in both the GM and WM regions. This could indicate neuronal degeneration and reduced WM integrity. While the GM is generally higher in volume than WM in adult brains, we postulate that degeneration of GM and WM combined is the cause for the differences in delusional severity. This also provides a new way to measure neurodegeneration of the brain.

The regression model controlled for a number of factors that were found to be associated with NABM abnormalities, including demographic factors such as age and sex, CSF tau, and genetic factors such as APOE4 (heterozygote). While some of these factors, such as age and sex, are not surprising, and consistent with the increased prevalence of GM and WM changes with age [25] and among females [26], the associations with APOE44 (homozygotes) and CSF tau were of particular interest, suggesting these factors may interact with sociodemographic factors to modulate damage to white and gray matter structures [27]. APOE44 (homozygotes) was significantly related to all biomarkers, as was CSF tau, with the exception of the damage biomarker. Both APOE4 (heterozygote) and tau have independently been linked to regional WM disruption [13] and GM disruption [28,29,30]. There was also a significant interaction between delusional severity and tau burden, consistent with prior literature suggesting a putative role for tau in mediating psychosis in dementia [12] and providing further evidence that tau’s role may be even more prominent in patients with more severe psychopathology. CSF tau is a microtubule-associated protein located in neuronal axons [31], and increased CSF tau levels are related to axonal loss [32] and neuronal degeneration [31]. As was shown, the NABM volume and integrity biomarkers in particular had significant interactions between the highest delusional severity and CSF tau, suggesting tau may play a role in disrupting the structural integrity of the NABM of delusional subjects. Moreover, the association of NABM biomarkers and CSF tau with severe delusions provides a potential mechanistic explanation in which volumetric and texture changes may underlie the development of severe delusions. NABM biomarkers may provide a surrogate marker for delusions and CSF tau, which could be used for disease monitoring and determining interventional time points.

Overall, our findings provide further evidence suggesting that psychotic symptoms including delusions may develop as a result of neurodegeneration in the GM and WM. Our results confirm that among cognitively impaired older adults, greater severity of delusions is associated with more significant changes in NABM volume, integrity and damage relative to patients with delusions of lesser severity. This has implications for clinical practice as it suggests that patients with more severe (and perhaps more persistent) delusions have greater levels of overall brain damage and thus should be investigated more rigorously for the presence of a neurodegenerative disorder. Moreover, it provides a potential explanation for the elevated mortality risk associated with the use of antipsychotic medications which have also been shown to accentuate cardiovascular risk burden through their metabolic effects [33]. In our sample, delusional severity was relatively evenly distributed among the diagnostic subgroups of cognition. Thus, it is unlikely that the results observed were attributable to differences in cognitive diagnosis or severity of cognitive impairment. In previous works, textural abnormalities in the brain were shown to distinguish between subjects who were cognitively intact and patients with MCI and AD [34]. Other studies in patients with vascular disease have also shown that texture abnormalities are frequently associated with decreased cognitive performance, executive dysfunction and conversion to dementia [35]. Few studies to date have examined texture abnormalities in patients with psychotic disorders such as schizophrenia, in which delusions are a common manifestation [27], one of the few published studies suggesting GM texture is better able to distinguish patients from controls than abnormalities in white-matter texture. Since the GM is generally higher in volume in adult brains, the NABM biomarkers may be dominated by changes in the GM. As the tau protein is related to tubule formation in the GM, the association with tau pathology and the imaging biomarkers further corroborates this finding.

Our study suggests NABM volume, integrity and damage may be useful surrogate markers of delusional severity and can provide quantitative metrics related to important sub-visual clues of dysfunction. While our study revealed a number of conclusions, there are some limitations that should be mentioned. We combined patients with MCI and AD, although there is likely an overlap. The sample size of this study was relatively small since there were few patients in ADNI that had all clinical variables and FLAIR MRI in the study. To combat this and try to balance group sizes, a post hoc analysis was completed which examined mild delusions and severe delusions (NPQ-I scores of 2 and 3, respectively) and similar results were found. Lastly, delusional severity was derived from NPI-Q scores and patients did not undergo a full psychiatric assessment. In addition, we did not include a non-delusional control cohort for comparison but hope to do so in future works.

## 5. Conclusions

Our study shows severity of delusions in older patients with early stage cognitive decline correlates with abnormalities in the NABM as described by volume, integrity and damage markers measured from FLAIR MRI. This suggests that not only the previously demonstrated GM degeneration [8], but also possibly WM degeneration, may play a crucial role in driving the development of delusions, and suggests a putative link between delusional severity and accelerated neurodegeneration. In addition to these findings, the tau protein was shown to be related to biomarkers and delusions, which suggest that tau may be mechanistically involved in these associations. APOE4 also had significant relationships with the NABM biomarkers. Studies on larger cohorts are required to further validate our findings.

## Figures and Tables

**Table 1:** Regression analysis comparing NABM biomarkers across delusional severity (1, 2, 3) and covariates. Bold is significant with *p* < 0.05.

	NABM Volume (%)		Integrity		Damage	
	Coefficient	*p*-Value	Coefficient	*p*-Value	Coefficient	*p*-Value
Delusion 2	−0.98%	0.52	−1.84	**0.03**	298.50	0.30
Delusion 3	−9.36%	**<0.01**	−3.71	**<0.01**	927.90	**0.01**
Tau	−0.01%	**0.03**	−0.006	**<0.01**	0.96	0.06
Age	−0.33%	**<0.01**	0.11	**<0.01**	39.89	**<0.01**
Gender	−2.37%	**<0.01**	−1.08	**<0.01**	403.00	**<0.01**
Education	−0.21%	0.07	−0.04	0.52	2.51	0.91
ApoE4	0.46%	0.46	0.43	0.25	−243.60	**0.04**
ApoE44	1.69%	**0.04**	1.18	**0.01**	−384.70	**0.01**
Delusion 2*Tau	0.001%	0.78	0.004	0.22	−0.63	0.48
Delusion 3*Tau	0.02%	**<0.01**	0.01	**<0.01**	−1.77	**0.03**

**Table 2:** Regression analysis comparing NABM biomarkers across mild- and severe-delusion groups and covariates. Bold is significant with *p* < 0.05.

	NABM Volume (%)		Integrity		Damage	
	Coefficient	*p*-Value	Coefficient	*p*-Value	Coefficient	*p*-Value
Severe Delusions	−3.72%	**<0.01**	−2.50	**<0.01**	485.40	0.05
Tau	−0.01%	**0.02**	−0.56	**<0.01**	1.03	**0.04**
Age	−0.30%	**<0.01**	−0.09	**<0.01**	37.90	**<0.01**
Gender	−1.79%	**<0.01**	−0.94	**<0.01**	363.60	**<0.01**
Education	−0.09%	0.42	−0.02	0.75	−7.04	0.91
ApoE4	0.40%	0.54	0.38	0.29	−244.10	**0.04**
ApoE44	2.26%	**0.01**	1.30	**<0.01**	−427.70	**<0.01**
Severe Delusions * Tau	0.01%	**0.01**	0.56	**<0.01**	−1.04	0.12

## Data Availability

Data are available from the first author upon reasonable request.

## Appendix A

**Table A1:** Image acquisition parameters for the multicenter FLAIR MRI delusional dataset.

Vendors	TR (ms)	TE (ms)	TI (ms)	Pixel Size (mm)	Slice Thickness (mm)	Magnetic Field (T)
GE, Siemens, Philips	9000–11002	90–153.978	2250–2500	0.8594–0.9375	5	3

**Table A2:** Composition of the experimental cohort.

Diagnosis	MCI *n* = 24	AD *n* = 17	MCI → AD *n* = 1
Age: MEAN ± SD	71.4 ± 7.52	80.2 ± 6.72	76.42 ± 0.49
Sex [nM:nF]	15:10	10:7	1:0
Delusional Severity: MEAN ± SD	1.40 ± 0.69	1.53 ± 0.74	2.0 ± 0

**Table A3:** Abbreviation table.

Abbreviation	Definition
NABM	Normal Appearing Brain Matter
GM	Grey-Matter
WM	White-Matter
WML	White-Matter Lesion
APOE4	Apolipoprotein epsilon4 allele
CSFTAU	Cerebro-Spinal Fluid (CSF) tau
AD	Alzheimer’s Disease
MCI	Mild Cognitive Impairment
NPI-Q	Neuropsychiatric Inventory Questionnaire
NPS	Neuropsychiatric Symptoms
FLAIR MRI	Fluid-attenuated inversion recovery (FLAIR) Magnetic resonance imaging (MRI)
ADNI	Alzheimer’s Disease Neuroimaging Initiative
